# Fat from dairy foods and ‘meat’ consumed within recommended levels is associated with favourable serum cholesterol levels in institutionalised older adults

**DOI:** 10.1017/jns.2019.5

**Published:** 2019-03-21

**Authors:** Yusi Liu, Shirley Poon, Ego Seeman, David L. Hare, Minh Bui, Sandra Iuliano

**Affiliations:** 1 Department of Endocrinology, University of Melbourne/Austin Health, Heidelberg, VIC 3084, Australia; 2 Centre for Ageing, Australian Catholic University, Melbourne Campus, Fitzroy, VIC 3065, Australia; 3 Department of Cardiology, University of Melbourne/Austin Health, Heidelberg, VIC 3084, Australia; 4 Melbourne School of Population and Global Health, University of Melbourne, Parkville, VIC 3010, Australia

**Keywords:** Aged-care, CVD, Dairy foods, Dietary fat, Meat food group, Saturated fat, HDL-C, HDL-cholesterol, LDL-C, LDL-cholesterol, TC, total cholesterol

## Abstract

CVD is common in older adults. Consumption of ‘meat’ (beef, pork, lamb, game, poultry, seafood, eggs) and dairy foods (milk, cheese, yoghurt) is encouraged in older adults as these foods provide protein and nutrients such as essential fatty acids, Ca, Fe, Zn and vitamins A, D and B_12_ required for healthy ageing. However, these foods also contain saturated fats considered detrimental to cardiovascular health. To determine the effect of their consumption on CVD risk we assessed associations between fat intake from ‘meat’ and dairy foods and serum cholesterol levels in 226 aged-care residents (mean age 85·5 years, 70 % female). Dietary intake was determined over 2 d using visual estimation of plate waste. Fat content of foods was determined using nutrition analysis software (Xyris, Australia). Fasting serum total cholesterol (TC), LDL-cholesterol and HDL-cholesterol were measured, and the TC:HDL-cholesterol ratio calculated. Associations were determined using random-effect models adjusted for CVD risk factors using STATA/IC 13.0. Total fat and saturated fat from ‘meat’ and dairy foods were associated with higher serum HDL-cholesterol levels, and dairy fat intake and number of servings were associated with a lower TC:HDL-cholesterol ratio. Every 10 g higher intake of fat and saturated fat from dairy products, and each additional serving was associated with a −0·375 (95 % CI −0·574, −0·175; *P* = 0·0002), a −0·525 (95 % CI −0·834, −0·213; *P* = 0·001) and a −0·245 (95 % CI −0·458, −0·033; *P* = 0·024) lower TC:HDL-cholesterol ratio, respectively. Provision of dairy foods and ‘meat’ in recommended amounts to institutionalised older adults potentially improves intakes of key nutrients with limited detriment to cardiovascular health.

CVD remains a leading cause of death in developed countries, with CHD and stroke accounting for 80 % of all CVD deaths^(^^1^^)^. In Australia, CVD accounts for the highest health care expenditure, with the greatest expenditure for people aged 65 years and over, at AU$3923 million annually (about US$3100 million, about €2600 million)^(^^2^^)^.

Modifiable risk factors for CVD include abnormalities in serum cholesterol levels, high blood pressure, diabetes, obesity and tobacco use, with current guidelines emphasising dietary and lifestyle changes in high-risk populations^(^^3^^)^. However, within the aged-care setting, these guidelines can be difficult to follow by individual institutionalised older adults as they have little autonomy over food provision and preparation and the variety of lifestyle options offered. Moreover, minimal nutritional standards are not mandated so food provision is at the discretion of the provider and is, in most cases, not meeting the recommended dietary guidelines^(^^4^^–^^6^^)^.

Dietary saturated fats from animals, certain plants and manufactured foods such as crisps, cakes and biscuits probably increase the risk of CHD due to their elevating effect on serum total cholesterol (TC) and LDL-cholesterol (LDL-C) levels, potentially contributing to the development of atherosclerotic plaque^(^^7^^,^^8^^)^.

The Australian Dietary Guidelines recommend that women and men over the age of 70 years consume daily: 2 and 2·5 servings of foods from the ‘meat’ food group (lean beef, pork, lamb, game, and fish/seafood, poultry, eggs, tofu nuts/seeds) and 4 and 3·5 servings of foods from the ‘dairy’ food group (milk, cheese, yoghurt and/or alternatives, mostly reduced fat), respectively, as part of a healthy diet, as these foods provide protein and key nutrients, such as iodine, Fe, Zn, vitamin B_12_ and essential fatty acids (‘meat’) and Ca, iodine, riboflavin, vitamins A, D and B_12_, and Zn (dairy products) to support healthy ageing. Consumption of these foods at the recommended levels ensures an adequate intake of these nutrients. Furthermore, the Guidelines recommend that for older adults >70 years of age consumption of non-core ‘discretionary’ foods (e.g. crisps, cakes, biscuits) be limited to no more than 2 and 2·5 servings daily for women and men, respectively, as these foods are high in saturated fat, added sugars and salt, or alcohol^(^^9^^)^.

The CVD risk associated with consumption of foods in the ‘meat’ food group is inconsistent, possibly due to the varying fatty acid profiles of, and constituents in, the various protein-rich foods contained in this food group^(^^10^^)^. For example, fish contains more of the amino acid taurine while red meat contains more methionine, the former protecting against and the latter exacerbating the development of atherosclerosis in animal models^(^^11^^)^. Dairy foods also have inconsistent effects on CVD outcomes, perhaps due to proteins, vitamins, minerals and other fatty acids in dairy products potentially offsetting deleterious effects of SFA via mechanisms such as interference of fat and cholesterol absorption^(^^12^^–^^15^^)^. Substitution of a diet high in refined carbohydrates and processed foods with a diet including both dairy foods and lean meats, such as the DASH (Dietary Approaches to Stop Hypertension) diet, resulted in reductions in blood pressure, serum cholesterol and CVD risk^(^^16^^)^.

Given the various fatty acid profiles and constituents in foods contained in these food groups we hypothesised that in institutionalised older adults, a higher consumption of servings of and fats from ‘dairy’ foods would be associated with lower serum cholesterol levels, a higher consumption of servings and fats from discretionary foods would be associated with elevated serum cholesterol levels and no association would be observed between the number of servings, or fat content of foods from the ‘meat’ food group and serum cholesterol levels.

## Methods

### Study population

Aged-care facilities in Melbourne and regional Victoria, Australia, were recruited between November 2013 and May 2014, as part of a cluster-randomised placebo-controlled trial. The inclusion criteria were: facilities were accredited with the Australian Aged-Care Quality Agency, and that accommodated predominantly ambulant residents. At baseline, informed consent was obtained from 354 residents (69·8 % females) aged 53–100 years to undergo anthropometric, dietary and nutrition assessments, and provide a fasting blood sample of which complete serum cholesterol profiles were available for 226 participants. These baseline data are reported in the present cross-sectional analysis. The overall study was approved by the Human Research Ethics Committee of Austin Health (project no. 04958) and was registered with the Australian and New Zealand Clinical Trials Registry (ACTRN 12613000228785).

### Assessment of food intake

Food provided is cooked fresh on the premises and follows a 4-week menu cycle. Meals consist of a continental-style (occasionally hot) breakfast, a midday meal offering a choice between two hot dishes, and dessert, an evening meal consisting of soup and either a hot or cold food option and dessert, and three snacks daily. Guidelines for foods provision are relatively structured, e.g. meat and at least two types of vegetables offered as a hot meal option, but may vary in their content, i.e. the type of meat and vegetables offered^(^^17^^)^.

Food intake was determined by visual estimation of plate waste^(^^18^^)^. Dietitians observed food consumption for each resident across the entire day from breakfast through to evening snacks if consumed, on two random days. A seven-point scale was used to represent the amount of food waste; 0 = no food remaining; +M = mouthful remaining; 1/4 = ¼ remaining; 1/2 = ½ remaining; 3/4 = ¾ remaining; –M = 1 mouthful consumed; 1 = no food eaten. The relative sizes of the meals served were compared with a weighed standard meal (denoted medium), and recorded as small (75 %), medium (100 %), large (125 %), or extra-large (150 %). All components of standard serves were weighed on a digital food scale (±1 g) (Sohnele Page Profi). This method is appropriate in this setting as it does not rely on recall or recording of foods by individuals in whom cognitive impairment is prevalent^(^^6^^)^.

Mean consumption of energy, macronutrients (total fats, saturated fats, monounsaturated fats, polyunsaturated fats, protein, carbohydrate), dietary fibre and minerals (Ca, Na) from all foods was calculated using Foodworks Version 7 (Xyris Software). Food composition values were derived from product-specific nutritional information on packaging. Missing nutrient values were obtained from NUTTAB 2010^(^^19^^)^.

The Australian Guide to Healthy Eating is a food selection guide consisting of five food groups: fruit; vegetables; grains; dairy products; and ‘meats’. Serving sizes are standard, and recommended number of servings dependent on an individual's age and sex, which if consumed in the suggested quantities ensures adequate intake of essential nutrients for good health^(^^9^^)^. Foods belonging to the ‘dairy product’ (milk, yoghurt, cheese and/or Ca-fortified plant milks) and ‘meat’ (lean beef, lamb, pork, game, and poultry, fish/seafood, eggs, tofu, nuts/seeds) food groups, and those classified as ‘discretionary’ (e.g. butter, cream, ice cream, pastries, processed meats, cakes, biscuits, spreads, soft drinks) were identified, but analysis limited to only dairy-based foods (no restriction on fat content) within the ‘dairy’ food group and animal-based foods within the ‘meat’ food group. Ca-fortified plant milks (dairy food group) and tofu and nuts/seeds (‘meat’ food group) were excluded from analyses. However, no residents consumed Ca-fortified plant milks or tofu, but some consumed nut/seed paste (e.g. peanut butter) as a spread on toast. The majority of dairy products were full fat, in line with the Australian Guidelines that suggest whole milk is appropriate for older adults, unless medically advised otherwise. Reduced- and low-fat dairy products are infrequently used in the aged-care setting and are generally only provided to specific residents as required. Total servings per d of each food group were calculated using serving sizes defined by the Australian Guide to Healthy Eating. One serving of ‘meat’ equalled 65 g of cooked meat, 80 g of cooked chicken, 100 g of canned fish, or two eggs. One serving of dairy food equated to 250 ml fresh or reconstituted powdered milk, 120 ml evaporated milk, 40 g hard cheese, 120 g ricotta cheese, or 200 g yoghurt. One serving of discretionary food provided 600 kJ/serving, and was equivalent to one tablespoon (20 g) butter, two tablespoons (40 g) cream, two scoops (75 g) ice-cream, 60 g pastries, two slices (60 g) processed meat, one slice (45 g) cake, or two to three (40 g) biscuits^(^^9^^)^.

### Covariates

Body weight of residents was obtained from facility documentation of monthly measured weight. Ulna length (UL) was used to estimate height using the following equation: males, height (cm) = 4·605UL + 1·308Age + 28·003; females, height (cm) = 4·459UL + 1·315Age + 31·485, with previously demonstrated high accuracy (*R*^2^ 0·96 and *R*^2^ 0·94, respectively)^(^^20^^,^^21^^)^. UL has been validated for use in elderly populations as it is less affected by ageing than standing height^(^^20^^,^^22^^)^. BMI was calculated using the equation weight (kg)/height^2^ (m^2^). Smoking status was categorised into (1) non-smoker/ex-smoker, or (2) current smoker. Medical history and medication use were recorded for each resident from medical records held at the facilities. CVD was defined as a diagnosis of CHD, cerebrovascular accident, transient ischaemic attack, peripheral artery disease and/or heart failure.

### Biochemistry

Fasting morning blood samples were collected from residents at each facility by a registered pathology service (Melbourne Pathology Inc.). Blood samples were analysed for serum TC, LDL-C and HDL-cholesterol (HDL-C), using a modular analyser (Cobas 8000 C701; Roche Diagnostics) (CV 1–5 %). TC:HDL-C ratio was generated as an additional variable using the equation TC (mmol/l)/HDL-C (mmol/l), as it is suggested to be a more sensitive and specific CVD risk predictor compared with TC alone^(^^23^^)^.

### Statistical analysis

Statistical analyses were conducted using STATA/IC 13.0. In this analysis dairy products were limited to milk, cheese and yogurt, and foods of animal origin in the ‘meat’ food group were analysed as continuous variables. Summary statistics are presented as means and standard deviations for continuous variables and sample sizes and percentages for categorical variables. The relationships between risk factors of fat (total and saturated) from dairy food and ‘meats’, and number of servings consumed, and the outcome variables of serum TC, HDL-C and LDL-C levels and the TC:HDL-C ratio were examined using random-effect models. This method takes into account correlation within each aged-care facility for an outcome variable.

Analyses were performed initially for each covariate to examine confounding effects. The covariates selected for this study were sex, age, BMI, diagnosis of CVD (yes/no), history of diabetes (yes/no), smoking status (current smoker, yes/no), fish consumption (yes/no), total energy, and PUFA and MUFA intake. Physical activity was not considered as this population is generally very sedentary and alcohol intake not considered as it was infrequently consumed. Those covariates with a *P* value less than 0·05 were chosen to adjust for the univariate relationship between a risk factor and an outcome. Those significant univariate relationships were then examined in a multivariate model, but separately for total fat, saturated fat and type of servings. *P* values <0·05 were deemed statistically significant.

## Results

Characteristics of participants are presented in Table 1. Total fat intake was 64 (sd 21) g/d (11·5 (sd 6·8) g/d from the dairy food group, 10·7 (sd 8·7) g/d from the ‘meat’ food group and 29·3 (sd 14·4) g/d from discretionary foods), while saturated fat intake was 30 (sd 10) g/d (7·2 (sd 4·3) g/d from the dairy food group, 3·9 (sd 3·3) g/d from the ‘meat’ food group and 14·4 (sd 6·9) g/d derived from discretionary foods). Mean number of servings was 1·4 (sd 0·8) from the dairy food group, 1·2 (sd 0·8) from the ‘meat’ food group and 4·7 (sd 1·1) for discretionary foods. The majority of participants did not achieve the recommended intake of servings from the dairy product (98 %) and ‘meat’ (85 %) food groups, and exceeded the recommended intake for discretionary foods (88 %). Individual foods within the ‘dairy product’ and ‘meat’ food groups were consumed in varying amounts. All residents consumed discretionary foods.

**Table 1.** Characteristics of Australian elderly aged-care residents (Mean values and standard deviations; numbers and percentages)

	Mean	sd
Sex		
Female *n* %	159 70·4	
Male *n* %	67 29·6	
Age (years)	85·7	7·7
BMI (kg/m^2^)	25·7	4·9
Smoking		
Current smoker *n* %	12 5·3	
Ex-smoker *n* %	54 23·9	
Non-smoker *n* %	160 70·8	
CVD *n* %	111 49·1	
IHD *n* %	64 28·3	
Cerebrovascular accident *n* %	38 16·8	
Cholesterol medication *n* %	82 36·3	
Statins *n* %	78 34·5	
Serum		
Cholesterol (mmol/l)	4·67	1·13
HDL-C (mmol/l)	1·41	0·42
LDL-C (mmol/l)	2·53	1·03
TC:HDL-C ratio	3·63	1·48
TAG (mmol/l)	1·60	0·71
Nutrients*		
Total energy intake (kJ/d)	6605	1510
Dairy products (servings/d)	1·4	0·8
Consumed milk *n* %	212 94	
Consumed cheese *n* %	86 38	
Consumed yoghurt *n* %	67 30	
Meeting recommended intake *n* %	5 2	
Meat (servings/d)	1·2	0·8
Consumed red meat/poultry *n* %	201 89	
Consumed fish *n* %	90 40	
Consumed eggs *n* %	90 40	
Meeting recommended intake *n* %	34 15	
Discretionary intake (servings/d)	4·7	1·1
Consumed discretionary foods *n* %	226 100	
Meeting recommended intake *n* %	27 12	
Total fat (g/d)	66·4	20·6
Dairy products (g/d)	11·7	6·6
Meat (g/d)	10·7	8·7
Discretionary foods (g/d)	29·3	14·5
Saturated fat (g/d)	30·6	9·2
Dairy products (g/d)	7·4	4·3
Meat (g/d)	3·9	3·3
Discretionary foods (g/d)	14·4	6·9
Polyunsaturated fat (g/d)	7·3	3·2
Monounsaturated fat (g/d)	19·0	6·1
Protein (g/d)	55·6	15·6
Carbohydrate (g/d)	192·7	51·6
Sugars (g/d)	99·0	34·3
Dietary fibre (g/d)	19·4	7·1
Ca (mg/d)	695	276
Meeting recommended intake *n* %	20 9	
Na (mg/d)	2073	897

HDL-C, HDL-cholesterol; LDL-C, LDL-cholesterol; TC, total cholesterol.

* Recommended servings for: women = dairy foods (4), ‘meat’ (2), discretionary foods (0–2); men = dairy foods (3·5), ‘meat’ (2·5), discretionary foods (0–2·5). One serving of dairy foods = 250 ml fresh or reconstituted powdered milk, 120 ml evaporated milk, 40 g hard cheese, 120 g ricotta cheese, 200 g yoghurt. One serving of ‘meat’ = 65 g of cooked meat, 80 g of cooked chicken, 100 g of canned fish, or two eggs. One serving of discretionary foods (600 kJ/serving) = one tablespoon (20 g) butter, two tablespoons (40 g) cream, two scoops (75 g) ice-cream, 60 g pastries, two slices (60 g) processed meat, one slice (45 g) cake, or two to three (40 g) biscuits^(^^9^^)^.

Associations between serum cholesterol levels and potential confounders are presented in Table 2. Smoking and higher BMI were associated with higher TC:HDL-C ratios, while diagnosis of CVD and use of cholesterol-lowering medications were associated with lower TC and LDL-C levels. No associations were observed between serum measures of cholesterol and dietary factors.

**Table 2.** Univariate analysis assessing association between each serum cholesterol measure and potential confounders (Coefficients with their standard errors)

	TC			HDL-C			LDL-C			TC:HDL-C ratio		
Variables	Coefficient	se	*P*	Coefficient	se	*P*	Coefficient	se	*P*	Coefficient	se	*P*
Female	0·47	0·14	0·001	0·27	0·06	<0·001	0·14	0·12	0·224	−0·46	0·24	0·052
Age*	−0·56	1·19	0·640	0·84	0·38	0·028	−1·08	1·13	0·336	−3·38	1·70	0·046
BMI*	−3·05	1·18	0·009	−2·95	0·61	<0·001	−1·61	1·08	0·137	5·08	1·73	0·003
CVD	−0·51	0·19	0·007	−0·05	0·06	0·434	−0·44	0·16	0·005	−0·19	0·22	0·395
Diabetes	−0·16	0·23	0·491	−0·06	0·07	0·410	−0·20	0·20	0·309	0·10	0·28	0·724
CLM	−1·12	0·13	<0·001	−0·07	0·05	0·186	−1·03	0·13	<0·001	−0·75	0·21	<0·001
Smoker	0·55	0·30	0·069	−0·21	0·11	0·055	0·57	0·29	0·054	1·41	0·57	0·013
Fish consumer*	0·57	15·6	0·971	7·75	6·49	0·233	0·68	15·6	0·965	−22·5	22·5	0·318
Total energy†	−0·22	0·48	0·656	−0·29	0·17	0·079	0·10	0·44	0·823	0·60	0·72	0·404
PUFA*	1·45	1·89	0·445	−1·01	0·85	0·237	2·41	1·74	0·164	3·85	3·36	0·252
MUFA*	0·97	1·14	0·395	−0·40	0·47	0·402	1·26	1·11	0·260	2·38	1·70	0·162

TC, total cholesterol; HDL-C, HDL-cholesterol; LDL-C, LDL-cholesterol; CLM, cholesterol-lowering medication; Smoker, current smoker.

* Estimated regression coefficient and standard error were multiplied by 100.

† Estimated regression coefficient and standard error were multiplied by 10 000.

After adjusting for covariates (Table 2), significant relationships were observed between total and saturated fat from ‘dairy products’ and ‘meat’ and HDL-C levels (Table 3). Every 10 g higher intake of fat and saturated fat from dairy foods was associated with a 0·103 (95 % CI 0·0018, 0·189) mmol/l (*P* = 0·018) and a 0·153 (95 % CI 0·018, 0·288) mmol/l (*P* = 0·026) higher HDL-C level, respectively. Corresponding associations from ‘meat’ were a 0·050 (95 % CI 0·005, 0·096) mmol/l (*P* = 0·030) and a 0·161 (95 % CI 0·027, 0·295) mmol/l (*P* = 0·018) higher HDL-C level, respectively. Higher dairy total fat, saturated fat and number of servings were associated with a lower TC:HDL-C ratio. Every 10 g higher intake of fat and saturated fat from dairy products, and each additional serving was associated with a –0·375 (95 % CI −0·574, −0·175) (*P* = 0·0002), a −0·525 (95 % CI −0·834, −0·213) (*P* = 0·001) and a −0·245 (95 % CI −0·458, −0·033) (*P* = 0·024) lower TC:HDL-C ratio, respectively. No associations were observed between fat from dairy foods, meat or discretionary foods, or servings of these foods and TC and LDL-C levels. In the multivariate analyses including fat (model I) and saturated fat (model II), similar associations remained for dairy products and ‘meat’ total and saturated fat and serum HDL-C, and dairy fat and saturated fat and the TC:HDL-C ratio (Table 4).

**Table 3.** Association between total dietary fat and saturated fat intake and fat and saturated from meat, dairy products and non-core discretionary foods and serum cholesterol levels, adjusted for significant covariates in Table 2* (Coefficients and 95 % confidence intervals)

	TC			HDL-C			LDL-C			TC:HDL-C ratio		
	Coefficient	95 % CI	*P*	Coefficient	95 % CI	*P*	Coefficient	95 % CI	*P*	Coefficient	95 % CI	*P*
Total fat	1·67	−0·39, 0·72	0·557	1·77	0·09, 3·44	0·039	1·06	−3·54, 5·67	0·651	−3·94	−9·89, 2·00	0·194
Saturated fat	1·20	−11·9, 14·3	0·858	4·95	0·32, 9·58	0·036	−0·90	−11·7, 9·90	0·870	−15·5	−30·5, –0·58	0·042
Dairy total fat	−10·0	−30·6, 10·5	0·339	10·3	1·77, 18·9	0·018	−14·0	−30·2, 2·19	0·090	−37·5	−57·4, –17·5	0·0002
Dairy saturated fat	−13·4	−45·3, 18·4	0·409	15·3	1·84, 28·8	0·026	−20·0	−44·8, 4·80	0·114	−52·4	−83·4, –21·3	0·001
Dairy servings	−47·5	−242, 147	0·633	73·7	−12·2, 160	0·093	−89·7	−237, 57·6	0·233	−245	−458, –32·5	0·024
Meat total fat	4·11	−7·93, 16·1	0·503	5·02	0·49, 9·56	0·030	0·09	−11·7, 11·9	0·987	−13·9	−28·4, 0·69	0·062
Meat saturated fat	16·5	−20·0, 53·0	0·375	16·1	2·74, 29·5	0·018	3·14	−29·8, 36·1	0·852	−41·8	−84·6, 0·92	0·055
Meat servings	62·4	−69·3, 194	0·353	26·1	−34·7, 86·9	0·400	33·2	−83·6, 150	0·578	−63·3	−257, 130	0·522
Discretionary total fat	2·47	−6·48, 11·4	0·589	0·48	−4·25, 5·22	0·842	2·86	−4·36, 10·1	0·437	−1·05	−15·1, 13·0	0·884
Discretionary saturated fat	5·19	−14·3, 24·6	0·601	2·61	−6·80, 12·0	0·586	5·65	−10·3, 21·6	0·487	−7·91	−37·1, 21·3	0·596
Discretionary servings	12·7	−56·8, 82·2	0·720	5·60	−20·3, 31·5	0·671	0·76	−56·3, 57·9	0·979	−9·18	−110, 92·1	0·859

TC, total cholesterol; HDL-C, HDL-cholesterol; LDL-C, LDL-cholesterol.

* All estimated regression coefficients and lower and upper CI values were multiplied by 1000.

**Table 4.** Multivariate analysis assessing association between total fat (model I) or saturated fat (model II) and HDL-cholesterol (HDL-C) or total cholesterol (TC):HDL-C ratio, adjusted for age, sex, BMI and cholesterol-lowering medications* (Coefficients and 95 % confidence intervals)

	HDL-C			TC:HDL-C ratio		
	Coefficient	95 % CI	*P*	Coefficient	95 % CI	*P*
Model I: total fat						
Dairy total fat	0·173	0·036, 0·310	0·013	−0·027	−0·042, −0·012	0·0003
Meat total fat	0·106	0·018, 0·194	0·018	−0·009	−0·019, 0·001	0·085
Model II: saturated fat						
Dairy saturated fat	0·174	0·037, 0·312	0·013	−0·039	−0·062, −0·016	0·001
Meat saturated fat	0·135	0·041, 0·229	0·005	−0·029	−0·059, 0·0003	0·053

* All variables were standardised to have mean zero and standard deviation of 1.

## Discussion

In institutionalised older adults, within recommended intake levels, higher intakes of fat or saturated fat from dairy products or ‘meat’ were associated with higher HDL-C levels but only dairy total fat, saturated fat and number of servings were associated with a lower TC:HDL-C ratio. Total or saturated fat from discretionary foods, or number of servings were not related to serum cholesterol levels.

The TC:HDL-C ratio is used as an atherogenic index, a lower ratio indicating lower risk^(^^24^^)^. A higher dairy fat intake was associated with a lower TC:HDL-C ratio, probably driven by the higher HDL-C observed with increasing dairy fat intake^(^^25^^)^. Significant increases in HDL-C levels have been observed in randomised studies of dairy food supplementation using cheese or probiotic yoghurt in healthy adults^(^^26^^,^^27^^)^. Sphingolipids from dairy fat and bacterial cell walls are thought to play a role in raising HDL-C, which has been observed in women when supplemented with fermented dairy products^(^^28^^)^. Dairy supplementation using probiotic yoghurt is also associated with reductions in TC in healthy, mildly to moderately hypercholesterolaemic individuals, and those with type 2 diabetes, in whom significant reductions in LDL-C were also observed^(^^27^^,^^29^^,^^30^^)^.

The mechanism of the lipid-lowering potential of dairy products is not well understood, but may relate to production processes or to a combination of constituents found in dairy foods. Dairy products fermented by some strains of lactic acid bacteria bind directly to bile acids in the gut thereby interfering with cholesterol absorption; while bifidobacteria species bind to, and uptake cholesterol particles in the presence of bile salts^(^^13^^)^.

Milk, cheese and yoghurt are good sources of Ca, which is known to act as a lipid-soluble salt, binding to fatty acids and bile acids in the small intestine, forming insoluble emulsifications, preventing absorption of fat, resulting in increased faecal fat and bile acid excretion^(^^12^^,^^31^^,^^32^^)^. A meta-analysis of fifteen randomised controlled trials that examined the effect of dairy products (or Ca) on faecal fat excretion estimated a 5·2 g increase in faecal fat excretion with the consumption of about 1200 mg of Ca daily^(^^12^^)^. Soerensen *et al*.^(^^33^^)^ observed in healthy adult males that the increase in serum TC and LDL-C levels when consuming a high-saturated fat diet were attenuated during a 2-week milk- or cheese-based diet relative to the isoenergetic control diet, and LDL-C decreased with increasing faecal fat excretion (*r* −0·404; *P* = 0·002).

Whey proteins have been shown to increase postprandial plasma amino acid levels and slow gastric emptying, leading to increased satiety^(^^34^^)^. Pal *et al*.^(^^35^^)^ reported a 7 % reduction in both TC and LDL-C compared with baseline, after a 12-week intervention using 54 g/d of whey supplements in eighty-nine overweight and obese individuals.

As participants consumed different combinations and amounts of milk, cheese and yoghurt, the magnitude and direction of changes to HDL-C, LDL-C or TC levels may also be differentially influenced by the diverse fatty acid profiles contained within dairy foods^(^^36^^)^. Therefore, the favourable cholesterol-altering effects of milk, cheese and yogurt may be via some, all, or a combination of mechanisms. In addition, the purported anti-inflammatory properties of some constituents of dairy foods, especially fermented dairy produce, may also help to reduce CVD risk^(^^37^^)^. However, fermented dairy products were consumed by approximately one-third of older adults in this cohort, so numbers were probably insufficient to enable detection of relationships between the number of servings and fat content of fermented dairy foods and serum cholesterol levels.

A favourable association between consumption of fat from the ‘meat’ food group and serum HDL-C levels was observed. Despite the varying types of foods in this food group, similar responses to serum cholesterol levels have been observed with consumption of red or white meat. For example, Maki *et al*.^(^^38^^)^, in their meta-analyses of randomised controlled trials investigating changes to serum cholesterol levels in response to beef (‘red’ meat) compared with poultry/fish (‘white’ meat) consumption, detected no differences to serum TC, LDL-C or HDL-C levels between the ‘red’ and ‘white’ meat types. Others have confirmed this observation^(^^39^^,^^40^^)^. However, a more favourable increase in serum HDL-C levels has been observed when fatty fish (e.g. cod and salmon) is consumed compared with lean fish and chicken, probably due to their fatty acid profile, but fatty fish is infrequently consumed in institutionalised older adults^(^^41^^)^.

Cardioprotective qualities have been reported with the consumption of *n*-3 fatty acids found in fish^(^^42^^)^. Howe *et al*.^(^^10^^)^ reported that, in addition to fish and seafood, meat (red meat, poultry and game) is also a major source of *n*-3 fatty acids (contributing to 48 and 43 % of daily *n*-3 fatty acid intake, respectively). Therefore, any deleterious effect of saturated fats in meat may be offset by its *n*-3 fatty acid content.

Previous dietary recommendations suggested that egg consumption be limited because of the high cholesterol content of eggs; however, recent evidence demonstrated increases in serum HDL-C as well as LDL-C with egg consumption. Moreover, eggs are also a rich source of xanthophyll carotenoids, which have been shown to have anti-inflammatory activities^(^^43^^–^^45^^)^. The number of servings of foods from the ‘meat’ food group consumed by residents was within the recommended guidelines of 2–2·5 servings daily, so within recommended levels these foods had a positive effect on serum HDL-C levels.

There are limitations in this study. First, food intake was recorded on randomly selected days, limiting the ability to investigate the relationship between individual foods within a food group and serum cholesterol levels. Second, participants were of advanced aged. An inverse relationship between serum cholesterol and age in the very old may result from selective survival^(^^46^^,^^47^^)^. However, given the ageing of the global population, the need for institutionalised care will increase, so these findings indicate that food provision (and consumption) in this setting may influence CVD risk. Furthermore, the observations from this cohort may relate to older adults of a similar age residing in the community as mean dietary dairy product intake in this cohort was compatible with the national average of 1·2 servings daily for those greater than 70 years of age^(^^48^^)^.

In summary, within the recommended intake levels, higher intakes of fat from dairy product and ‘meat’ sources were associated with higher HDL-C levels and higher dairy food consumption was associated with a lower TC:HDL-C ratio, which are predictive of a more favourable cardiovascular risk profile.

### Conclusion

In institutionalised older adults, consumption of foods from the dairy product and ‘meat’ food groups within recommended amounts has the potential to improve intakes of key nutrients from these food groups with limited detriment to cardiovascular health.
